# Occupational Stress and Mental Health Among Healthcare Workers Serving Socially Vulnerable Populations During the COVID-19 Pandemic

**DOI:** 10.3389/fpubh.2021.782846

**Published:** 2021-12-09

**Authors:** V. Nelly Salgado de Snyder, Alice P. Villatoro, Marisol D. McDaniel, Ana Sofia Ocegueda, Deliana Garcia, Deborah Parra-Medina

**Affiliations:** ^1^National Institute of Public Health, Center for Health Systems Research, Cuernavaca, Mexico; ^2^Latino Research Institute, College of Liberal Arts, The University of Texas at Austin, Austin, TX, United States; ^3^Santa Clara University, Program of Public Health, Santa Clara, CA, United States; ^4^Migrant Clinicians Network, Austin, TX, United States

**Keywords:** occupational stress, healthcare workers, health providers, COVID-19, mental health, anxiety, depression, vulnerable groups

## Abstract

The purpose of this study was to analyze occupational and personal stressors, mental health indicators, perceived discrimination and help-seeking behaviors among healthcare workers and providers (HCWPs) serving socially vulnerable groups such as immigrants, refugees, farmworkers, homeless individuals, people living in poverty, and other disadvantaged populations in the United States (U.S.) during the COVID-19 pandemic. Using a cross-sectional descriptive approach, we gathered information between July and September 2020, from a sample of 407 affiliates of two national organizations of clinic-based HCWPs who worked at federally funded and community safety-net clinics. Informed consent was obtained from all participants who completed a self-administered online survey available in English and Spanish. Our results indicated that the HCWPs serving vulnerable groups in the midst of the pandemic experienced high levels of occupational and personal stressors as well as anxiety and depressive symptomology. Major occupational stressors were excessive workload, long working-hours, and institutional barriers to refer and follow-up on their clients' access to needed social services. High-rated personal stressors included sleep disorders, lack of and child-care, partner's loosing job, and other family related situations. Our findings suggest that HCWPs working with vulnerable populations need specialized interventions that bolster their mental health and well-being as the pandemic continues to unfold. We recommend implementing initiatives that encourage HCWPs' to be actively involved in clinic decisions regarding employee safety and protection as well as in management decisions to improve work place infrastructure and capacity to respond to the social needs of their clients. Lessons learned from the pandemic are useful tools in designing protocols for addressing the mental-health needs of HCWPs in health-care organizations that attend to socially underprivileged populations.

## Introduction

Coronavirus disease 2019 (COVID-19) has generated international concern as vaccination efforts continue worldwide and contagion rates persist with new variants endangering the lives of millions. Global social disparities and their underlying social determinants have exacerbated during the pandemic, striking harder on socially marginalized groups, leaving them exposed and frequently unprotected, from a deadly virus (1–3). As reported in studies conducted pre-COVID-19 pandemic, caring for underprivileged populations who have been systematically excluded from society and healthcare systems imposes an additional burden on the well-being and mental health of healthcare providers, many of whom feel a loss of control at work and an inability to help their vulnerable clients (4–6).

Since COVID-19 was declared a pandemic in March 2020, healthcare workers and providers (HCWPs) worldwide have operated under unprecedented pressure to contend with the influx of both types of patients in healthcare facilities: those infected with SARS-Cov-2 and others presenting a diversity of health ailments not directly related to the new virus. International studies conducted during the pandemic in hospital and clinical settings have found that the increased workload, shortage of personal protective equipment (PPE)—especially at the beginning of the health emergency—and heightened risk of exposure have caused many HCWPs to experience significant levels of stress, sleep disturbances, and burnout (7–12). International systematic literature reviews and other original research reports have consistently found that COVID-19 represents a risk factor for stress, depression, anxiety, and post-traumatic stress disorder (PTSD) among healthcare personnel (13–17). Other recent studies reported that HCWPs are more vulnerable to psychological distress manifested in high levels of uncertainty, insecurity, depression, stress, anxiety, anger, fear, insomnia, and PTSD (11, 12). Furthermore, poor sleep quality occurs nearly twice as frequently among HCWPs as it does in the general population, most likely due to sleep disturbances, which have also been linked to depression and distress (9). Nurses and those who work more closely and for more extended periods with COVID-19 patients seem to be one of the most affected groups (7, 18).

Despite its importance, only recently was a global call made to health systems and leaders to protect the mental health of HCWPs, as long-term exposure to COVID-19 and their clients is a risk factor affecting both their mental health and quality of services they provide (19–21). Furthermore, organizations of healthcare professionals in the United States (U.S.) and worldwide have drawn attention to clinicians' mental health and other HCWPs during the pandemic (22–25).

Research conducted prior to the COVID-19 pandemic suggests that HCWPs assisting populations living in socially vulnerable conditions often feel anguished at being unable to provide appropriate and sufficient resources to the population they serve because a number of their clients do not qualify for certain services as they lack medical insurance, documentation to reside in the U.S., or may have English language limitations (4, 5, 26). In addition, HCWPs are at risk of burn-out, which has been recently reconceptualized by the World Health Organization (27) as a condition resulting from being exposed to chronic stress in the workplace that has not been successfully managed. Its three major characteristics are feelings of energy depletion, increased mental distance from one's job, or feelings of negativism or cynicism related to one's job, and reduced professional efficacy. Burn-out is not classified as a medical condition, but it is included in the 11th Revision of the International Classification of Diseases (ICD-11) as an occupational phenomenon (27). Burn-out has been reported as a common problem among health and social service providers along with other mental health affections, such as secondary trauma and compassion fatigue, from listening to the traumatic experiences of the vulnerable groups they serve. For example, a study examining rates of secondary trauma among caregivers working with Mexican and Central American refugees revealed that more than half experienced emotional numbness, trouble sleeping, intrusive thoughts, and irritability (5). Another study on the mental health of front-line workers serving homeless populations, identified feelings of helplessness and frustration by their perceived inability to improve the situation of their patients and a high prevalence of burnout, traumatic stress, and diminished compassion satisfaction (i.e., inability to derive innate positive feelings from helping others) (6).

To mitigate the adverse effects of stress and protect themselves from burnout and secondary trauma, HCWPs seek support from family and friends who are regarded as essential resources for self-care, they also engage in physical activities and favorite pastimes (4, 28). Another way of coping is to increase the distance between themselves and their patients by “shutting down” their emotional responses (5, 6). It must be noted that not all HCWPs look for professional help in times of crisis due to a number of reasons that include among others, feeling that they can cope alone with the problems they face, social stigma, and personal, financial and institutional barriers to access needed mental health services (28).

To our knowledge, little research exists on how the COVID-19 pandemic accentuated occupational stressors and undermined mental health of HCWPs based in community clinics serving socially vulnerable groups. In this study, conducted amid the COVID-19 pandemic, we sought to describe and assess the occupational and mental health challenges faced by HCWPs attending to the healthcare needs of migrants, refugees, farmworkers, homeless individuals, people living in poverty, and other socially vulnerable groups in the U.S.

## Materials and Methods

We collected original data through a web-based cross-sectional study of clinic-based HCWPs affiliated with two national organizations in the U.S. serving migrants and other socially vulnerable populations. All affiliates to these two organizations work at federally funded and community safety-net clinics (Federally Qualified Health Centers-FQHC) that provide health care to uninsured individuals regardless of their ability to pay. We sent affiliate members email invitations to participate in our research that included information about the purpose and procedures of the study as well as a link to the brief online survey. The invitation was distributed through the listserv of each organization, comprising HCWPs, administrators and advocates. Inclusion criteria for participation included having worked in a healthcare setting for at least two weeks prior to the survey and having interacted with patients in one of the following healthcare roles: case manager/case coordinator, behavioral health worker, healthcare provider, community health worker, outreach worker, patient navigator, medical assistant, certified nurse assistant, nurse specialist, dietitian or clinical pharmacist. Informed consent was obtained electronically prior to accessing the survey link. Every seven days for up to four weeks, we sent follow-up email reminders to listserv recipients regarding study eligibility and participation. Upon survey completion, we sent participants a small electronic monetary incentive ($5 USD). Data was collected from July 1, 2020 to September 14, 2020. The project was submitted to and approved by the Institutional Review Boards (IRBs) of the organizations involved.

In total, 801 respondents clicked on the link to the Qualtrics survey, with 551 consenting to complete the survey (68.7%). There was some overlap in listserv recipients across both partner organizations; as such, a participant could complete the survey more than once. Duplicates were rare (*n* = 33 of 801 or 4.1%) and removed by verifying participant email addresses and birthdates. Because the organizational listservs included HCWPs who did not work directly with patient populations, it was impossible to calculate a response rate. Among the 768 non-duplicate participants who started the survey, 518 were eligible and consented to participate in the study. In the end, 407 completed the majority of the survey (79.0%).

The online survey was especially created for this study by designing our own questions and drawing on items from existing studies. The 15-min survey was available in English and Spanish and was self-administered using Qualtrics, a web-based survey platform. It included questions on sociodemographic characteristics, self-rated health and mental health symptoms, stress, substance use, COVID-19-related occupational and personal stressors, perceived discrimination and coping mechanism such as help-seeking behaviors and self-care practices during the pandemic.

Participants were asked to rate their overall physical and mental health (1 = Excellent to 5 = Poor) using two separate questions. We assessed mental-health symptoms using the Patient Health Questionnaire for Depression and Anxiety (PHQ-4), a brief screening tool that measures burden of depressive and anxiety symptoms (29). Participants were asked how frequently in the past 14 days they were bothered by specific symptoms such as (1) feeling nervous, anxious or on edge, (2) not being able to stop or control worrying, (3) feeling down, depressed or hopeless and (4) having little interest or pleasure in doing things (ranging from 0 = Not at all to 3 = Nearly every day). The PHQ-4 results in two subscales, anxiety (items 1 and 2) and depression (items 3 and 4) symptoms. The subscales have good internal consistency with Cronbach's alpha values of 0.84, and 0.81, respectively. Prior research suggests that a total score of 3 or greater on these subscales identifies potential cases of anxiety and depression (29, 30).

Substance use during the previous seven days was measured using the Substance Use questions of the Understanding America Study—Coronavirus in America COVID Survey (31). Participants were asked to report the number of days they drank alcohol, used cannabis products, consumed other recreational drugs, smoked cigarettes, or used electronic cigarettes (e.g., vape pen). We calculated the average number of days per week each participant consumed these substances (range: 0–7 days and created a dichotomous variable using scores of at least one SD above the sample mean (0 = Low, 1 = High).

We assessed perceived stress in the 14 days using the Perceived Stress Scale-4 (PSS-4) (32). Items included: (1) being unable to control important things, (2) feeling confident about handling personal problems, (3) feeling things were going their way, and (4) difficulties were piling up (0 = Never to 4 = Very Often). Items 2 and 3 were reverse coded. A total score was calculated for each participant, with a higher score suggesting higher perceived stress (alpha = 0.61). We created a separate dichotomous variable that specified whether a participant scored at least one SD above the sample mean (0 = Low, 1 = High).

Based on the available literature regarding the impact of COVID-19 on HCWPs (7–9, 22, 23), we developed two short scales specifically for this study, that measured occupational and personal stressors associated with the COVID-19 pandemic (response options ranged from 0 = Not stressful at all, to 5 = Very stressful) during the 14 days prior to the survey. The occupational and personal stressors scales were constructed based on face validity and demonstrated good internal consistency (alpha = 0.88 for occupational stressors and alpha = 0.78 for personal). Occupational stressors were 11 items and included: long working hours, work overload, interpersonal problems with co-workers, communicating bad news to patients and their family members, inability to communicate directly with patients due to cultural and language barriers, scarcity of PPE, fear of bringing the virus home, voluntary isolation from the family, lack of proper safety protocols at the clinic, inability to connect patients with appropriate social services (e.g., food bank, rent or legal assistance), and lack of resources to follow-up on patients after visit. Personal stressors included six items: insufficient sleep, employment loss by their partner/spouse, insomnia, immigration problems related to oneself and/or family members, and lack of proper child care arrangements. For each stressor scale, we calculated the mean score. We also created separate dichotomous variables indicating whether or not participants experienced high levels of stress (at least one SD above the sample mean for each scale: 0 = Low level of stress and 1 = High level of stress).

Perceived discrimination among CHWPs was also assessed in our study, which had been previously studied in the USC national survey (31). This is another form of tension experienced by HCWPs, particularly because of disinformation at the beginning of the pandemic (33, 34) and because it coincided with a period of time where social division prevailed in the U.S. Participants were asked whether or not they had experienced discrimination in the last 14 days using items especially developed for our study based on face validity and to identify their perceptions for their feeling discriminated against (e.g., race/ethnicity, immigrant status and their role as a healthcare worker) during the pandemic: people treated them less courteously, they were provided poorer service at restaurants and stores, people were afraid of them, or subjected them to threats/harassment (No = 0 or Yes = 1). We counted the number of perceived discriminatory experiences reported by participants.

Finally, respondents were asked to reflect on their coping responses such as mental health help-seeking and self-care behaviors during the pandemic. We asked HWCPs if they sought support from a mental-health professional in person and/or virtually during the pandemic (0 = No, 1 = Yes). Likewise, HWCPs reported on the number of days over the past seven days they engaged in the following self-care behaviors: practicing meditation, exercising, finding time to relax, and socializing with family and friends either online or in person. We computed the mean number of days engaged in all self-care behaviors. We also constructed a dichotomous variable for the level of self-care behaviors the participant engaged in (0 = None or few, 1 = High—at least one SD above the sample mean).

We report descriptive statistics including means, SDs and percentages with 95% confidence intervals to examine self-rated health, mental health indicators (anxiety and depressive symptomology, substance use, and stress), occupational and personal stressors, perceived discrimination, help-seeking, and self-care behaviors of HCWPs during the COVID-19 pandemic. All descriptive estimates were adjusted for age.

Additionally, a series of multivariable regression analyses (e.g., linear, logistic, or count regressions depending on the outcome variable of interest) were performed to examine how the demographic characteristics of HCWPs (i.e., age, gender, Latinx identity, marital status, family size, and educational attainment) and type of healthcare provider (e.g., community health worker, behavioral health provider etc.) were associated with mental health, occupational stressors, and well-being outcomes (see Appendices A–E in Supplementary Material). There were no statistically significant differences across type of healthcare providers for the outcomes of interest, with the exception of help-seeking behaviors and potential exposure to COVID-19 (these results are highlighted in the results section). For parsimony, we present the multivariable analyses that only include the demographic characteristics. Both descriptive analyses (*n* = 407) and multivariable regressions (*n* = 387) presented only use complete cases.

## Results

### Sociodemographic Characteristics of Participants

Table 1 presents the sociodemographic characteristics of the study participants. Most respondents were women, who self-identified as Latinx, living with a partner, with a mean age of 44.40 years, had earned an undergraduate degree, and performed duties of community health workers. The majority of participants worked in clinics located mostly in the Southwestern states of the U.S. such as Texas, California, Arizona and Colorado.

**Table 1:** Sociodemographic characteristics of total sample (*N* = 407).

**Participant Characteristics**	**Total Sample (*N* = 407)**
	**% or Mean (SD)**
Age (19–79 range)	44.40 (13.09)
Participants 50+ years old	33.66%
Female	86.88%
Latinx	75.43%
Married/living with partner	65.36%
Family size	2.45 (1.69)
Highest Level of Education	
Undergraduate degree	27.52%
Graduate degree	40.79%
Primary Health Care Role	
Community health worker	49.39%
Health provider (nurse, physician)	19.41%
Behavioral health provider	12.29%
Case manager/Case coordinator	11.06%
Medical assistant	6.88%
Dietician	0.98%
State of residency	TX, CA, AZ, CO, VA, GA, PR

### Self-Rated Health, Anxiety and Depression Symptoms, Substance Use, and Perceived Stress

Table 2 presents the age-adjusted results obtained for the patterns of mental health symptoms, stress and substance use among HCWPs. A small proportion of respondents appraised their general physical and mental health conditions as “poor to fair” (8.78 and 13.00%, respectively). In all cases, they rated their mental health as being worse than their physical health. Although the HCWPs had experienced low levels of anxiety and depressive symptoms, one-quarter reported high levels of anxiety symptoms, while 13.39% experienced high depressive symptoms—that is, they met the threshold of likely having an anxiety or depressive disorder. Thus, a sizeable proportion of HCWPs were at high-risk for mental health problems related to anxiety and depression. Most respondents in this high-risk group were women, under 50 years of age, had a graduate degree, and had been working longer shifts during the pandemic. The PSS-4 had an age-adjusted perceived stress mean value of 5.64, with 14.32% of respondents experiencing high levels of stress. Substance use was generally low among HCWPs, with participants consuming alcohol, cigarette products and marijuana less than once a week, on average. Approximately one in 10 reported using substances more than one day per week.

**Table 2:** Age-adjusted means, standard deviation, and percentages for self-rated health, symptoms of anxiety and depression, substance use, and perceived stress (*N* = 407).

	**Mean (SD)**	**95% CI**	**High Outcome %**
Self-Rated health (1 = Excellent to 5 = Poor)			
Physical health	2.36 (0.09)	[2.28, 2.44]	8.78%^a^
Mental health	2.46 (0.26)	[2.37, 2.56]	13.00%^a^
PHQ-4 (Total score range: 0–6)			
Anxiety subscale	2.01 (0.48)	[1.84, 2.17]	25.67%^b^
Depression subscale	1.35 (0.32)	[1.20, 1.49]	13.39%^b^
Substance use scale (# days/week)	0.40 (0.05)	[0.34, 0.46]	12.48%^c^
PSS-4 Perceived Stress Score			
(Total score range: 0-16)	5.64 (0.40)	[5.37, 5.94]	14.32%^c^

a *Percent of participants in the total sample who reported fair or poor self-rated health.*

b *Percent of participants in the total sample reporting a total score ≥ 3.*

c *Percent of participants in the total sample reporting a score at one or more standard deviations above the sample mean*.

Multivariable linear regressions were conducted to examine how the demographic characteristics of HCWPs were related to the continuous mental health outcomes (Appendices A,B in Supplementary Material). Net of the covariates, married/cohabiting HCWPs compared to those never married reported lower ratings of self-rated physical and mental health—that is, they indicated better physical and mental health (Appendix A in Supplementary Material). In general, older HCWPs were protected against anxiety and depressive symptoms and substance use than younger HCWPs. Latinx vs. non-Latinx HCWPs reported fewer days of using substances during the pandemic (Appendix B in Supplementary Material). In contrast, males and those with dissolved marriages (e.g., divorced, widowed) reported more frequent substance use than females and the never married, respectively. No demographic characteristics were related to perceived stress.

Similar patterns were observed for high risk of mental health problems (Appendix C in Supplementary Material). Logistic regressions examined how the demographic characteristics were associated with fair/poor self-rated physical and mental health, risk of anxiety and depressive problems, frequent substance use, and high perceived stress. Older vs. younger ages, being married or divorced/separated/widowed compared to never married, and identifying as Latinx vs. not were protective against fair/poor self-rated mental health (only for age), high anxiety, high depression (only for age), and substance use (Latinx identity only; Appendix C in Supplementary Material). Larger family size was also protective of frequent substance use. In contrast, larger family size and having high educational attainment (a bachelors or graduate degree vs. high school or less) were associated with higher risk of anxiety problems. HCWPs with dissolved marriages were four times more likely to engage in frequent substance use than HCWPs who have never been married (Appendix D in Supplementary Material). No demographic characteristics were associated with high perceived stress.

### COVID-19 Potential Stressors and Perceived Discrimination

As indicated in Table 3, HCWPs reported higher mean stress levels associated with occupational than personal stressors. The occupational stressors with high stress ratings, scoring 4 and 5 (not shown in Table 3) were excessive workload (44.23%), long work hours (39.06%), fear of bringing the virus home (36.85%), lack of resources to follow-up with patients (35.62%), the inability to connect immigrant patients with needed social services such as food banks and assistance with rent or legal matters (34.65%). At least one-fourth of the respondents associated high stress levels with situations such as communicating bad news to their clients (26.04%) and lack of adequate PPE (25.06%). Interpersonal problems with other staff members and lack of proper safety protocols in their place of employment were also reported as very stressful by 22.36 and 22.85% of the HCWPs, respectively. Linear regressions revealed that males and HCWPs with high educational attainment experienced more job-related stressors than females and HCWPs with a high school education or less, respectively (Appendix E in Supplementary Material). Moreover, males and HCWPs with dissolved marriages were found to be nearly three times more likely than females and those never married to experience high job stressors, net of the covariates (Appendix F in Supplementary Material).

**Table 3:** Age-adjusted means, standard deviation, and percentages for COVID-19 related stressors, perceived discrimination, mental health help-seeking and self-care behaviors (*N* = 407).

	**Mean (SD)**	**95% CI**	**High Outcome %**
COVID-19 related stressors			
Occupational stressors	2.01 (0.08)	[1.93, 2.10]	17.64%^a^
Suspected contact with COVID patient			46.04%
Personal stressors	1.23 (0.06)	[1.15, 1.31]	13.61%^a^
Perceived discrimination			
Number of discriminatory events	0.59 (0.10)	[0.49, 0.67]	15.54%^a^
Treated with less courtesy (*n* = 151; no/yes)			72.23%^b^
Received poorer services (*n* = 151; no/yes)			26.46%^b^
Others were afraid of them (*n* = 151; no/yes)			43.04%^b^
Threatened or harassed (*n* = 151; no/yes)			17.19%^b^
Mental health help-seeking and self-care behaviors			
In-person mental health appointment (*n* = 406; no/yes)			7.39%^c^
On-line mental health appointment (*n* = 406; no/yes)			23.02%^c^
Self-care behaviors (# days/week; *n* = 406)	2.78 (0.41)	[2.64, 2.93]	14.29%^c^

a *Percent of participants in the total sample reporting a score at one or more standard deviations above the sample mean.*

b *Percentage for the type of discriminatory events participants experienced during the past 14 days, only among those that reported any discrimination.*

c *Percent of participants in the total sample who engaged in help-seeking or self-care behaviors*.

In regards to personal stressors with high-stress ratings (not shown in Table 3), insufficient sleep (39.06%), insomnia (21.14%), and job loss by a partner/spouse (15.23%) were the highest rated (scoring 4 and 5). Other stressors reported with high ratings by fewer respondents were not having proper childcare arrangements while working (8.81%), and concerns about family members' immigration status (9.58%). The multivariable analyses showed that Latinx HCWPs, being married, and family size were associated with more personal-related stressors during the pandemic (Appendix E in Supplementary Material). However, when examining risk of high personal stressors—that is, at least one standard deviation above the sample mean—Latinx HCWPs vs. not and higher family size were associated with greater odds of experiencing this high level of personal stressors (Appendix F in Supplementary Material).

Overall, more than one-third of the sample perceived at least one discriminatory event, with an average of experiencing almost two discriminatory events during the past 14 days. Nearly one in five HCWPs experienced high levels of discrimination (at least one SD above the sample mean). Among those perceiving some form of discrimination, the most common events reported were being treated less courteously or respectfully than others (72.23%) and being feared (43.04%). Experiencing these two events was most often attributed to their role as a HCWP (32.10 and 70.82%, respectively). In multivariable analyses, only males were found to experience greater counts of discriminatory events than women, controlling for the other demographic characteristics (Appendix E in Supplementary Material). However, these demographic characteristics were not significantly related to risk of experiencing high discrimination (Appendix F in Supplementary Material).

Having had direct contact with at least one patient diagnosed with or suspected of having COVID-19 was considered, in and of itself, an additional source of stress: nearly half of respondents indicated having had such contact. Male HCPWs were twice more likely than females to be potentially exposed to COVID-19 at their workplace (Appendix F in Supplementary Material). Other characteristics associated with increased odds of exposure included family size and higher educational attainment. In general, HCWPs that had a health provider role (e.g., doctor, nurse) were more likely to be potentially exposed than community health workers, behavioral health providers, and other HCWPs (not shown; results available upon request).

### Help-Seeking and Self-Care Behaviors

Most HCWPs reported having engaged in mental health help-seeking behaviors and self-care activities. While <10% sought mental-health support from a professional in person, nearly one-quarter did so virtually. Latinx HCWPs were more likely to seek in-person mental health support than non-Latinx HCWPs (Appendix G in Supplementary Material). No differences across demographic characteristics were found for remote mental health visits. However, case managers/coordinators and behavioral health providers had greater odds of seeking remote mental health support than traditional health providers (e.g., doctors, nurses), net of the demographic characteristics (not shown).

Participants reported engaging in self-care behaviors aimed at enhancing their well-being, such as meditation and physical exercise, on average almost three days a week. The most commonly performed self-care activity (i.e., behaviors practiced at least four days a week) included socializing with others whether virtually or in-person (52.3%), and taking time to relax (34.9%). Less than one-third of HCWPs engaged in meditation (23.9%) or physical exercise (29.2%) for at least four days a week. Overall, almost 15% practiced these behaviors more than four days a week. Controlling for the model covariates, the multivariable analyses demonstrated that older HCWPs reported more days of engaging in self-care behaviors than younger HCWPs (Appendix E in Supplementary Material). Additionally, behavioral health providers reported more self-care days than healthcare providers (not shown). Older HCWPs were 5% more likely to engage in frequent self-care activities (Appendix G in Supplementary Material). No other characteristics were associated with self-care.

## Discussion

The current study communicates findings on a very pertinent and timely issue that to our knowledge, has not been addressed in previous published research: the analysis of how attending socially vulnerable clients during the COVID-19 pandemic has undermined the well-being and mental health of HCWPs. The research reported in this study aims at describing the mental health indicators and occupational and personal stressors that HCWPs in the U.S. encountered during the initial months of the COVID-19 pandemic.

During the pandemic, HCWPs are considered frontline, essential, and critical infrastructure workers; and those providing services to vulnerable groups seem to be working under higher pressure and occupational stress because COVID-19 has hit the socially vulnerable harder than other populations. Socially disadvantaged groups in the U.S. have experienced disproportionate COVID-19 morbidity and mortality rates due to persistent inequities in underlying conditions such as wealth, poverty, employment, housing, health status, access to health care, and exposure to the virus related to occupation, among and others (35).

Our findings suggest that HCWPs serving vulnerable groups in the midst of the pandemic suffer adverse mental health repercussions that are reflected in a self-perception of poor mental health status, high levels of stress, and the manifestation of anxiety and depressive symptomatology possibly related to occupational and personal stressors. Research has shown that in general, one year into the pandemic, HCWPs are being confronted by feelings of anger, uncertainty, and insecurity in addition to sleeping disorders, anxiety, depression, grief, and even suicide [i.e., (13, 17, 36)]. In our study, participants with high scores of anxiety and depression symptoms are considered a high-risk group for developing serious mental health problems. This group at risk was comprised of mostly Latina women, under 50 years of age who worked longer shifts during the pandemic. Similar findings have been reported, for instance, in a study conducted in Mexico with a large sample of frontline health workers who reported clinically significant symptoms of anxiety, depression and somatization among female providers, under the age of 50 with long-exposure to COVID-19 patients (17).

HCWPs in our study experienced high levels of stress associated with occupational situations. They also perceived being the target of discrimination because of their role as healthcare providers. The high levels of stress associated with occupational situations could be related to the fact that the COVID-19 pandemic has exposed, exacerbated, and confirmed existing inequalities in society as well as unveiled new ones, placing socially vulnerable populations at greater risk during this health emergency [i.e. (37, 38)]. Serving high-risk populations, in and of itself, is a stressful job (38). Additionally, the longer than expected duration of the pandemic along with the impossibility to predict the end of this health crisis, has placed at stake the long-term mental well-being of health workers attending populations with high healthcare needs.

The personal stressors we identified with higher scores were mostly related to sleep disorders and concerns about nuclear and extended family members. The occupational and personal stressors seemed to potentiate each other to create an amplifying negative effect impacting on the HCWPs mental health. Other studies (11, 17) have reported similar findings of daily stress accumulation and consequential manifestation of psychological problems such as depressive symptoms, anxiety and other forms of mental discomfort, including suicidal ideation. Recent publications have documented that responding to the health needs of others during the pandemic has been consistently identified as a source of significant stress and a mental health challenge among HCWPs (1, 12). Many HCWPs find it difficult to work under extreme pressures such as deciding how to allocate limited resources to equally needy patients, how to find a balance between their own mental health care needs and those of their clients, and how to align their duty to patients with their own personal responsibilities to family and friends (12, 26). Listening to concerns and having compassion toward those more severely affected by the pandemic seems to lead to additional stress resulting in fear, anger, frustration, hopelessness, guilt, depression, and even suicidal ideation (36).

In our study, we also found that a small proportion of HCWPs, actively sought mental health resources in person or virtually to help them cope with their stressful occupational and personal situations. A larger number, however, reported emotional support and companionship from family and friends to try to find comfort and emotional well-being. Similar findings have been reported in previous research that emphasize the crucial supportive role of family and close friends as coping responses when facing stressful situations (4, 5, 39). Getting involved in activities known to help reduce stress, such as exercise and meditation, were reported only by a small proportion of our participants, perhaps due to their demanding work schedules and lack of time to attend both family- and job-related responsibilities. Our findings suggest the need to develop and implement strategic interventions to protect the mental health of HCWP working with socially disadvantaged groups, as these providers seem to use limited resources to reduce their emotional discomfort.

The World Health Organization (19) highlighted the importance of protecting the rights of HCWPs regarding working hours and the prevention of psychological distress, fatigue, occupational burnout, stigma and physical/psychological violence. Organizations of healthcare professional, alike, have pledged to implement specific measures to improve occupational safety and protect the physical and mental health of health workers (24, 25). However, focusing on the needs of patients affected with health problems derived from coronavirus and its variants, while at the same time continuing with vaccination-related actions (i.e., providing information and education on vaccines and actual immunizations) have increased the demands upon health personnel, making it difficult to lighten their workloads.

Preventing burnout and psychosocial problems among this essential group of healthcare workers require individual interventions and appropriate organizational policies, as well as infrastructure capable of meeting their needs. Moreover, supporting HCWP mental health requires developing a systemic, multi-level approach that includes access to a variety of individual- and group-level mental health interventions and a coordinated organizational response (20, 21). Professional associations, healthcare facilities and other employment sites should offer accessible in-person and virtual mental-health-crisis interventions for their staff using digital platforms, online networks and telemedicine communications. Though, it should be noted that not all HCWPs will have access to, or be willing to utilize mental-health services due to feeling thy can deal with their own problems, stigma, the cost of professional counseling, –given that some already face financial burdens associated to their partners' losing their employment. Nevertheless, healthcare organizations may help by making available brief self-care strategies and the use of rapid mental health screeners to self-monitor their well-being. Also important is to teach HCWPs new and efficient coping strategies to deal with stress and mental discomfort associated with both occupation and personal stressors. Organizations can also contribute to reducing the occupational stressors by for instance, facilitating a safe and healthy work environment with access to PPE and supplies, allocating additional time to rest and recuperate, stipulating clear communication lines, roles and expectations of all clinic staff members, and engaging HCPWs and other staff in decision-making that involves their personal safety.

Previous research shows that providers working with vulnerable populations (37) report high levels of stress primarily related to having insufficient institutional resources to care for their patients, which is consistent with our own findings. This is why a potential way to mitigate the occupational stress resulting from the pandemic is to assure that HCWPs have access to effective and efficient referral systems for their clients by improving their work place infrastructure and capacity to respond to the social needs of their patients (26). Working with the clinics' management on ways of improve services for their clients without compromising the mental health of HCWPs could prove a useful cost-effective strategy for occupational stress reduction.

This contribution has several limitations. First, it was a cross-sectional design with a modest non-representative sample of health workers, which posed some limitations on the scope of our analysis, our findings' generalizability and, more importantly, in establishing a cause-effect relationship. Second, all responses to the measures were self-reported by HCWPs and clinical diagnostic tools were not used to assess mental health status. However, our study utilizes robust mental health symptoms and distress measures that are highly correlated with mental disorders and have been validated for use with the general U.S. population. Third, we did not have a comparison group of HCWP not working with vulnerable populations that would allow us to analyze differences and similarities between the two groups of HCWPs. Finally, the survey did not include questions about the type of labor contract (i.e., fixed-term, permanent) HCWPs had with their employers, nor about commuting, which may have influenced their responses to the variables considered in this study.

Despite these limitations, this study makes a valuable contribution to the research literature because it provides baseline, descriptive data on the mental health, and stress levels of safety net providers, who have been likely dealing with the brunt of COVID-19 with their socially vulnerable clients since the initial months of the COVID-19 pandemic. The findings derived from this study could be of great value for designing protocols within healthcare organizations serving socially vulnerable populations post pandemic and for developing systemic, multi-level approach interventions to protect the mental health of HWPs.

Future research with health providers should consider paying more attention to specific determinants of mental health discomfort and the particular needs of HCWP working with the socially underprivileged. This often-overlooked group of professionals have not received the attention they deserve from policy makers, clinic managers, professional associations, and researchers given the conditions of scarcity they frequently face in their workplace. FQHC in the U.S. often receive limited funds to run their operations and must meet a number of stringent criteria to receive funding, and the lack of resources to help others seems to add pressure to their already demanding commitments to their clients. The growing body of literature focusing on the mental health of health providers must include comparative studies with HCWPs tending the underserved. Future projects also need to include longitudinal tracking of the social, organizational, and personal factors affecting the mental health of HCWPs as the pandemic continues to unfold worldwide. In closing, we want to emphasize that protecting the mental health of HCWPs is not only a necessary part of providing high-quality health care, but also a global priority and a moral obligation of health care leaders, health systems, and health organizations whose staff is exposed to COVID-19-related risks.

## Data Availability Statement

The raw data supporting the conclusions of this article will be made available upon request to the corresponding author.

## Ethics Statement

The studies involving human participants were reviewed and approved by the Institutional Review Board (IRB) at the University of Texas at Austin.

## Author Contributions

VS conceived the project and wrote the manuscript's original draft. AV and MM worked on the data analyses. AV, MM, DG, AO, and DP-M contributed to the writing of this manuscript. AV was affiliated to the Latino Research Institute of the University of Texas at Austin when data collection for this study was conducted. All authors read and approved the final version of this contribution.

## Funding

Funds for this research and for open access publication fees were provided by the Latino Research Institute at the University of Texas at Austin, through a Distinguished Research Fellowship awarded to VS.

## Conflict of Interest

The authors declare that the research was conducted in the absence of any commercial or financial relationships that could be construed as a potential conflict of interest.

## Publisher's Note

All claims expressed in this article are solely those of the authors and do not necessarily represent those of their affiliated organizations, or those of the publisher, the editors and the reviewers. Any product that may be evaluated in this article, or claim that may be made by its manufacturer, is not guaranteed or endorsed by the publisher.
