# Supplementary material for: Occupational Stress and Mental Health Among Healthcare Workers Serving Socially Vulnerable Populations During the COVID-19 Pandemic
Source: Front Public Health. 2021 Dec 9;9:782846. doi: 10.3389/fpubh.2021.782846 (PMC8695753; doi:10.3389/fpubh.2021.782846)
Supplement: Supplementary file 1 [file Table_1.DOCX]

**Supplementary Material**

**Appendix A.** Linear regression models examining differences in physical and mental health outcomes of HCWPs across demographic characteristics (n = 387)

|  | **Self-Rated**  **Physical Health** | | **Self-Rated**  **Mental Health** | | **Total Anxiety**  **Symptoms** | | **Total Depression Symptoms** | |
| --- | --- | --- | --- | --- | --- | --- | --- | --- |
|  | **b** | **[95% CI]** | **b** | **[95% CI]** | **b** | **[95% CI]** | **b** | **[95% CI]** |
| Age | 0.00 | [-0.01, 0.00] | -0.02*** | [-0.02, -0.01] | -0.03*** | [-0.05, -0.02] | -0.02*** | [-0.04, -0.01] |
| Gender |  |  |  |  |  |  |  |  |
| Female | ref |  | ref |  | ref |  | ref |  |
| Male | -0.07 | [-.033, 0.19] | 0.04 | [-0.24, 0.32] | -0.26 | [-0.78, 0.26] | 0.06 | [-0.40, 0.52] |
| Latinx Identity |  |  |  |  |  |  |  |  |
| No | ref |  | ref |  | ref |  | ref |  |
| Yes | 0.10 | [-0.12, 0.32] | -0.15 | [-0.39, 0.08] | -0.13 | [-0.56, 0.30] | 0.13 | [-0.26, 0.51] |
| Marital Status |  |  |  |  |  |  |  |  |
| Never Married | ref |  | ref |  | ref |  | ref |  |
| Married/Living with Partner | -0.27* | [-0.49, -0.04] | -0.34** | [-0.58, -0.09] | -0.33 | [-0.78, 0.12] | -0.04 | [-0.44, 0.35] |
| Separated/Divorced/Widowed | -0.19 | [-0.52, 0.14] | -0.15 | [-0.50, 0.20] | -0.48 | [-1.13, 0.18] | -0.09 | [-0.67, 0.49] |
| Family Size | 0.05 | [-0.01, 0.10] | 0.00 | [-0.06, 0.06] | 0.01 | [-0.10, 0.12] | -0.02 | [-0.12, 0.07] |
| Education |  |  |  |  |  |  |  |  |
| High School or less | ref |  | ref |  | ref |  | ref |  |
| Some college/AA | -0.02 | [-0.34, 0.30] | 0.17 | [-0.17, 0.52] | 0.38 | [-0.26, 1.02] | 0.26 | [-0.31, 0.82] |
| Bachelor's degree | -0.01 | [-0.30, 0.28] | 0.23 | [-0.08, 0.54] | 0.44 | [-0.13, 1.02] | 0.14 | [-0.37, 0.65] |
| Graduate degree | -0.27 | [-0.55, 0.01] | -0.02 | [-0.32, 0.29] | 0.55 | [-0.01, 1.11] | 0.12 | [-0.38, 0.62] |
| Constant | 2.64*** | [2.16, 3.13] | 3.47*** | [2.95, 3.99] | 3.45*** | [2.49, 4.41] | 2.23*** | [1.38, 3.08] |

NOTE: **p* < .05, ***p* < .01, ****p* < .001

**Appendix B.** Linear regression models examining differences in substance use and perceived stress of HCWPs across demographic characteristics (n = 387)

|  | **Days Engaged in Substance Use (n=387)** | | **Total Perceived Stress (n = 387)** | |
| --- | --- | --- | --- | --- |
|  | **b** | **[95% CI]** | **b** | **[95% CI]** |
| Age | -0.01* | [-0.01, 0.00] | -0.02 | [-0.05, 0.00] |
| Gender |  |  |  |  |
| Female | ref |  | ref |  |
| Male | 0.22* | [0.04, 0.40] | -0.47 | [-1.36, 0.43] |
| Latinx Identity |  |  |  |  |
| No | ref |  | ref |  |
| Yes | -0.41*** | [-0.56, -0.26] | -0.28 | [-1.03, 0.46] |
| Marital Status |  |  |  |  |
| Never Married | ref |  | ref |  |
| Married/Living with Partner | 0.06 | [-0.09, 0.22] | -0.34 | [-1.11, 0.44] |
| Separated/Divorced/Widowed | 0.27* | [0.05, 0.50] | -0.52 | [-1.64, 0.61] |
| Family Size | -0.04 | [-0.07, 0.00] | 0.05 | [-0.14, 0.23] |
| Education |  |  |  |  |
| High School or less | ref |  | ref |  |
| Some college/AA | 0.10 | [-0.12, 0.32] | 0.43 | [-0.67, 1.53] |
| Bachelor's degree | 0.08 | [-0.12, 0.27] | -0.09 | [-1.08, 0.90] |
| Graduate degree | 0.02 | [-0.18, 0.21] | -0.19 | [-1.16, 0.78] |
| Constant | 0.94*** | [0.61, 1.27] | 7.23*** | [5.58, 8.87] |

NOTE: **p* < .05, ***p* < .01, ****p* < .001

**Appendix C.** Logistic regression models examining differences in risk of poor physical and mental health outcomes of HCWPs across demographic characteristics (n = 387)

|  | **Fair/Poor Self-Rated Physical Health** | | **Fair/Poor Self-Rated Mental Health** | | **High Anxiety Symptoms** | | **High Depressive Symptoms** | |
| --- | --- | --- | --- | --- | --- | --- | --- | --- |
|  | **OR** | **[95% CI]** | **OR** | **[95% CI]** | **OR** | **[95% CI]** | **OR** | **[95% CI]** |
| Age | 0.98 | [0.95, 1.02] | 0.96** | [0.93, 0.99] | 0.97** | [0.95, 0.99] | 0.97** | [0.94, 0.99] |
| Gender |  |  |  |  |  |  |  |  |
| Female | ref |  | ref |  | ref |  | ref |  |
| Male | 0.65 | [0.19, 2.26] | 0.93 | [0.36, 2.42] | 0.62 | [0.28, 1.38] | 0.83 | [0.33, 2.10] |
| Latinx Identity |  |  |  |  |  |  |  |  |
| No | ref |  | ref |  | ref |  | ref |  |
| Yes | 1.10 | [0.42, 2.83] | 0.70 | [0.33, 1.45] | 0.82 | [0.46, 1.45] | 1.30 | [0.62, 2.76] |
| Marital Status |  |  |  |  |  |  |  |  |
| Never Married | ref |  | ref |  | ref |  | ref |  |
| Married/Living with Partner | 0.83 | [0.36, 1.96] | 0.71 | [0.36, 1.42] | 0.51* | [0.29, 0.90] | 1.13 | [0.55, 2.31] |
| Separated/Divorced/Widowed | 0.59 | [0.14, 2.53] | 0.77 | [0.24, 2.49] | 0.25* | [0.08, 0.75] | 0.51 | [0.13, 2.03] |
| Family Size | 1.00 | [0.81, 1.24] | 1.04 | [0.88, 1.23] | 1.16* | [1.00, 1.34] | 0.93 | [0.78, 1.12] |
| Education |  |  |  |  |  |  |  |  |
| HS or Less | ref |  | ref |  | ref |  | ref |  |
| Some college/AA | 2.50 | [0.62, 10.07] | 2.83 | [0.83, 9.61] | 1.85 | [0.66, 5.21] | 1.33 | [0.43, 4.11] |
| BA | 1.93 | [0.51, 7.24] | 2.44 | [0.77, 7.75] | 2.63* | [1.04, 6.66] | 1.27 | [0.46, 3.51] |
| Graduate degree | 1.17 | [0.30, 4.63] | 1.39 | [0.42, 4.61] | 2.92* | [1.15, 7.39] | 1.27 | [0.46, 3.50] |

NOTE: **p* < .05, ***p* < .01, ****p* < .001

**Appendix D.** Logistic regression models examining differences in risk of frequent substance use and high perceived stress of HCWPs across demographic characteristics (n = 387)

|  | **Frequent Substance Use** | | **High Perceived Stress** | |
| --- | --- | --- | --- | --- |
|  | **OR** | **[95% CI]** | **OR** | **[95% CI]** |
| Age | 0.98 | [0.95, 1.00] | 0.98 | [0.96, 1.01] |
| Gender |  |  |  |  |
| Female | ref |  | ref |  |
| Male | 2.89* | [1.22, 6.83] | 0.94 | [0.37, 2.37] |
| Latinx Identity | |  |  |  |
| No | ref |  | ref |  |
| Yes | 0.19*** | [0.09, 0.39] | 0.78 | [0.39, 1.54] |
| Marital Status | |  |  |  |
| Never Married | ref |  | ref |  |
| Married/Living with Partner | 1.57 | [0.63, 3.94] | 0.65 | [0.32, 1.31] |
| Separated/Divorced/Widowed | 4.42* | [1.35, 14.49] | 0.97 | [0.34, 2.81] |
| Family Size | 0.74* | [0.58, 0.94] | 1.02 | [0.86, 1.21] |
| Education |  |  |  |  |
| HS or Less | ref |  | ref |  |
| Some college/AA | 1.06 | [0.25, 4.41] | 1.50 | [0.41, 5.49] |
| BA | 0.91 | [0.26, 3.28] | 2.06 | [0.65, 6.57] |
| Graduate degree | 0.98 | [0.29, 3.34] | 2.17 | [0.69, 6.88] |

NOTE: **p* < .05, ***p* < .01, ****p* < .001

**Appendix E.** Linear regression models examining differences in job- and personal-related stressors, discrimination, and self-care activities of HCWPs across demographic characteristics

|  | **Job-Related Stressors (n = 387)** | | **Personal-Related Stressors (n = 387)** | | **Count of Discrimination Events (n = 387)^a^** | | **Days Engaged in Self-Care (n = 386)** | |
| --- | --- | --- | --- | --- | --- | --- | --- | --- |
|  | **b** | **[95% CI]** | **b** | **[95% CI]** | **IRR** | **[95% CI]** | **b** | **[95% CI]** |
| Age | -0.01 | [-0.01, 0.00] | 0.00 | [-0.01, 0.01] | 0.99 | [0.97, 1.00] | 0.03*** | [0.02, 0.04] |
| Gender |  |  |  |  |  |  |  |  |
| Female | ref |  | ref |  | ref |  | ref |  |
| Male | 0.33* | [0.06, 0.59] | 0.20 | [-0.04, 0.44] | 1.64* | [1.07, 2.51] | -0.08 | [-0.53, 0.37] |
| Latinx Identity |  |  |  |  |  |  |  |  |
| No | ref |  | ref |  | ref |  | ref |  |
| Yes | -0.10 | [-0.31, 0.12] | 0.21*** | [0.01, 0.41] | 1.08 | [0.73, 1.60] | 0.03 | [-0.34, 0.41] |
| Marital Status |  |  |  |  |  |  |  |  |
| Never Married | ref |  | ref |  | ref |  | ref |  |
| Married/Living with Partner | 0.18 | [-0.05, 0.41] | 0.29** | [0.08, 0.49] | 0.73 | [0.50, 1.06] | -0.34 | [-0.73, 0.05] |
| Separated/Divorced/Widowed | 0.11 | [-0.22, 0.44] | 0.06 | [-0.24, 0.36] | 0.92 | [0.51, 1.64] | -0.28 | [-0.89, 0.28] |
| Family Size | 0.04 | [-0.01, 0.10] | 0.08** | [0.03, 0.13] | 1.03 | [0.94, 1.13] | -0.07 | [-0.16, 0.02] |
| Education |  |  |  |  |  |  |  |  |
| High School or less | ref |  | ref |  | ref |  | ref |  |
| Some college/AA | 0.25 | [-0.08, 0.57] | 0.17 | [-0.12, 0.46] | 1.58 | [0.86, 2.90] | -0.22 | [-0.77, 0.33] |
| Bachelor's degree | 0.35* | [0.05, 0.64] | 0.18 | [-0.08, 0.44] | 1.54 | [0.87, 2.72] | 0.39 | [-0.10, 0.89] |
| Graduate degree | 0.31* | [0.02, 0.59] | -0.13 | [-0.39, 0.13] | 1.54 | [0.87, 2.72] | 0.19 | [-0.29, 0.68] |
| Constant | 1.78*** | [1.30, 2.27] | 0.69** | [0.25, 1.13] |  |  | 1.63*** | [0.80, 2.45] |

^a^ Negative binomial regression model was estimated; **p* < .05, ***p* < .01, ****p* < .001

**Appendix F.** Logistic regression models examining risk of high job- and personal-related stressors, discrimination, and exposure to COVID-19 of HCWPs across demographic characteristics

|  | **High Job Stressors** | | **High Personal Stressors** | | **High Discrimination** | | **Potential COVID-19 Exposure** | |
| --- | --- | --- | --- | --- | --- | --- | --- | --- |
|  | **OR** | **[95% CI]** | **OR** | **[95% CI]** | **OR** | **[95% CI]** | **OR** | **[95% CI]** |
| Age | 0.99 | [0.96, 1.01] | 0.99 | [0.96, 1.02] | 0.98 | [0.96, 1.01] | 1.01 | [0.99, 1.03] |
| Gender |  |  |  |  |  |  |  |  |
| Female | ref |  | ref |  | ref |  | ref |  |
| Male | 2.65** | [1.31, 5.35] | 1.88 | [0.84, 4.22] | 1.73 | [0.78, 3.83] | 2.19* | [1.15, 4.18] |
| Latinx Identity |  |  |  |  |  |  |  |  |
| No | ref |  | ref |  | ref |  | ref |  |
| Yes | 0.90 | [0.46, 1.74 | 2.85* | [1.04, 7.80] | 1.49 | [0.68, 3.27] | 0.75 | [0.45, 1.27] |
| Marital Status |  |  |  |  |  |  |  |  |
| Never Married | ref |  | ref |  | ref |  | ref |  |
| Married/Living with Partner | 1.62 | [0.76, 3.44] | 1.92 | [0.80, 4.63] | 0.61 | [0.30, 1.22] | 1.42 | [0.82, 2.46] |
| Separated/Divorced/Widowed | 2.87* | [1.03, 7.97] | 1.72 | [0.49, 6.04] | 0.99 | [0.35, 2.80] | 1.33 | [0.60, 2.96] |
| Family Size | 1.11 | [0.94, 1.30] | 1.26* | [1.06, 1.50] | 1.12 | [0.95, 1.32] | 1.17* | [1.03, 1.34] |
| Education |  |  |  |  |  |  |  |  |
| HS or Less | ref |  | ref |  | ref |  | ref |  |
| Some college/AA | 0.95 | [0.34, 2.61] | 1.22 | [0.45, 3.27] | 1.95 | [0.67, 5.69] | 2.44* | [1.06, 5.63] |
| BA | 1.71 | [0.70, 4.15] | 1.48 | [0.59, 3.68] | 1.79 | [0.65, 4.91] | 3.47*** | [1.62, 7.43] |
| Graduate degree | 1.07 | [0.44, 2.63] | 0.75 | [0.28, 1.96] | 1.44 | [0.52, 4.02] | 3.46*** | [1.63, 7.32] |

NOTE: **p* < .05, ***p* < .01, ****p* < .001

**Appendix G.** Logistic regressions examining differences in help-seeking behaviors and frequent self-care activities of HCWPs across demographic characteristics (n = 386)

|  | **In-Person Mental Health Visit** | | **Remote Mental Health Visit** | | **Frequent Self-Care** | |
| --- | --- | --- | --- | --- | --- | --- |
|  | **OR** | **[95% CI]** | **OR** | **[95% CI]** | **OR** | **[95% CI]** |
| Age | 1.01 | [0.98, 1.04] | 0.99 | [0.97, 1.01] | 1.05*** | [1.02, 1.07] |
| Gender |  |  |  |  |  |  |
| Female | ref |  | ref |  | ref |  |
| Male | 0.65 | [0.18, 2.35] | 0.70 | [0.31, 1.60] | 0.76 | [0.30, 1.87] |
| Latinx Identity |  |  |  |  |  |  |
| No | ref |  | ref |  | ref |  |
| Yes | 13.32* | [1.73, 102.28] | 1.81 | [0.95, 3.43] | 0.59 | [0.30, 1.15] |
| Marital Status |  |  |  |  |  |  |
| Never Married | ref |  | ref |  | ref |  |
| Married/Living with Partner | 0.61 | [0.23, 1.60] | 0.59 | [0.32, 1.08] | 1.10 | [0.50, 2.43] |
| Separated/Divorced/Widowed | 0.82 | [0.22, 3.08] | 0.80 | [0.33, 1.96] | 0.75 | [0.25, 2.20] |
| Family Size | 1.03 | [0.80, 1.31] | 0.93 | [0.80, 1.09] | 0.83 | [0.68, 1.03] |
| Education |  |  |  |  |  |  |
| HS or Less | ref |  | ref |  | ref |  |
| Some college/AA | 2.77 | [0.53, 14.51] | 1.23 | [0.43, 3.56] | 0.71 | [0.22, 2.32] |
| BA | 2.17 | [0.43, 10.83] | 2.07 | [0.83, 5.21] | 1.02 | [0.39, 2.68] |
| Graduate degree | 3.90 | [0.83, 18.41] | 2.33 | [0.93, 5.82] | 0.82 | [0.32, 2.11] |

NOTE: **p* < .05, ***p* < .01, ****p* < .001
